# Spatial autocorrelation analysis of health care hotspots in Taiwan in 2006

**DOI:** 10.1186/1471-2458-9-464

**Published:** 2009-12-14

**Authors:** Pui-Jen Tsai, Men-Lung Lin, Chien-Min Chu, Cheng-Hwang Perng

**Affiliations:** 1 College of Liberal and General Education, Aletheia University, Republic of China, Taiwan; 2 Department of Tourism, Aletheia University, Republic of China, Taiwan; 3 Graduate institute of Geography, National Taiwan University, Republic of China, Taiwan; 4 Department of Statistics and Actuarial Science, Aletheia University, Republic of China, Taiwan

## Abstract

**Background:**

Spatial analytical techniques and models are often used in epidemiology to identify spatial anomalies (hotspots) in disease regions. These analytical approaches can be used to not only identify the location of such hotspots, but also their spatial patterns.

**Methods:**

In this study, we utilize spatial autocorrelation methodologies, including Global Moran's I and Local Getis-Ord statistics, to describe and map spatial clusters, and areas in which these are situated, for the 20 leading causes of death in Taiwan. In addition, we use the fit to a logistic regression model to test the characteristics of similarity and dissimilarity by gender.

**Results:**

Gender is compared in efforts to formulate the common spatial risk. The mean found by local spatial autocorrelation analysis is utilized to identify spatial cluster patterns. There is naturally great interest in discovering the relationship between the leading causes of death and well-documented spatial risk factors. For example, in Taiwan, we found the geographical distribution of clusters where there is a prevalence of tuberculosis to closely correspond to the location of aboriginal townships.

**Conclusions:**

Cluster mapping helps to clarify issues such as the spatial aspects of both internal and external correlations for leading health care events. This is of great aid in assessing spatial risk factors, which in turn facilitates the planning of the most advantageous types of health care policies and implementation of effective health care services.

## Background

The Taiwan National Health Insurance (NHI) program was implemented in 1995. The coverage rate of the program has increased from 92.41% in 1995 to more than 96.16% in 2000. Coverage further increased to 98% after the inclusion of active military forces in 2001. At the beginning of 2004, NHI data related to medical care, such as the leading causes of death, were reclassified and reprocessed in relation to smaller units or areas (e.g., precincts or townships rather than the country as a whole). Regional data from the statistical analysis system (SAS) program are announced publicly by the NHI in regular annual reports (e.g., NHI, 2006) [1]. These reports provide an accurate and reliable data source to help investigators explore health care issues in Taiwan.

In the study of spatially-related objects or characteristics, one first describes the regional characteristics that differentiate areas one from another, and then proceeds with the analysis of spatial interrelations [2]. Common spatial techniques used in health research include disease mapping, clustering techniques, diffusion studies, identification of risk factors through map comparisons and regression analysis [3]. Spatial clustering techniques are important for statistical consideration, and form the beginning steps in the development of models for predicting disease risk sites. Disease risk sites are, specifically, areas located close to one another that tend to share similar disease risk factors, because they share similar environments and are also often connected by the spread of communicable disease via vectors or host dispersal [4].

Cuzick and Edwards (1990) proposed three general methodological approaches that can be utilized for the detection of clustering: the first is based on cell counts; the second on autocorrelative adjacencies of cells with high counts; and the third based on determining the distance between events [5]. Numerical methods have been extensively adopted for spatial cluster detection in health research and epidemiology, especially for the processing of areal data. The analytical approaches include the following: join-count statistics [6]; Ohno statistics [7]; Poisson statistics [8]; Global Moran's I [9-11]; Global Geary's *C *[9-11]; General Getis-Ord's G [12]; Local Moran's I [13]; and Local *Gi(d) *and Gi*(d) [12-14]. Spatial autocorrelation statistics such as the Moran's I and Geary's *C *methods are global, in the sense that they estimate the overall degree of spatial autocorrelation in a dataset. The possibility of spatial heterogeneity suggests that the estimated degree of autocorrelation may vary significantly across geo-space. Local spatial autocorrelation statistics provide estimates which are disaggregated to the level of the spatial analysis units, allowing assessment of dependency relationships in different areas. Local *Gi(d) *and Gi*(d) statistics can be used to make autocorrelation comparisons in different neighborhoods. A global average is used to help identify local regions of strong autocorrelation. Local version of the Moran's I and Geary's *C *statistics are also available.

In this study, we develop a method for ascertaining the spatial clustering associated with the 20 leading health care events, based on medical care data collected by the Taiwan NHI agency. Furthermore we also investigate potential spatial risks which contribute to these health care events and redefine epidemiologic and spatially referenced data.

## Methods

### Study area

**Figure 1:**
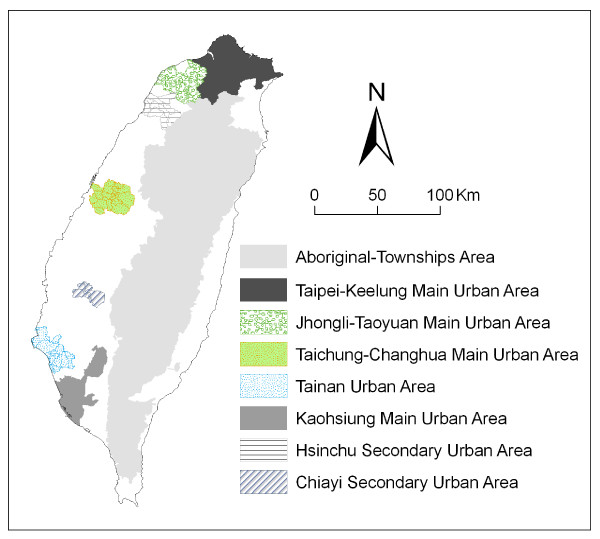
**Map of urban areas and aboriginal townships in the study area**. Map of the study area divided into 349 administrative districts including 7 urban areas and an integrated area of 29 aboriginal townships.

The study area includes the main island of Taiwan only (excluding all islets), comprising more than 22 million inhabitants in the year 2000, living in an area of 36,000 km^2^. There are a total of 349 local administrative government areas, which include 5 main urban areas, 2 secondary urban areas, 187 rural townships, and 29 aboriginal townships (Figure 1). According to a bulletin from the Ministry of Interior issued in 1996, urban areas are regions having at least one metropolitan center and can include neighboring cities and townships which share socioeconomic activities. Main urban areas are defined as those with a population larger than one million, specifically, Taipei-Keelung, Kaohsiung, Taichung-Changhua, Jhongli-Taoyuan and Tainan. Secondary urban areas are defined as those with a residential population ranging from 0.3 to 1 million (for example, Hsinchu and Chiayi).

### Data collection and management

The data were collected from contractual medical care institutions, which in this study, means institutions where the NHI covers the costs of prescription medicines and treatment at outpatient clinics. Such facilities accumulate detailed databases of medical costs for inpatient care. The number of outpatient cases were classified in relation to disease codes, as defined in the 1975 edition of "The International Classification of Diseases, 9th Revision, Clinical Modification" (hereafter, ICD 9 CM). Criteria for refining the data were first established. Some data were not included in the final statistical data set. For example, cases where patients suffer from diseases which defy code classification, mismatched ID numbers, and so on. Disease codes were classified by gender and age. Cases with the same ID numbers but different diseases were counted as different instances [1].

Medical care data obtained from the NHI, 2006 report were examined, and the prevalence rates of the 20 leading causes of death calculated. Diseases classifications (made according to the International Classification of Disease, Injuries, and Causes of Death, 1975) are indicated in parentheses. They include the following: malignant neoplasms (ICD 08-14); cerebrovascular disease (ICD 29); heart disease (ICD 250, 251, 27, and 28* which includes a partial listing of ICD 420-429); diabetes mellitus (ICD 181); accidents and adverse side effects (ICD E47-E53); pneumonia (ICD 321); chronic liver disease and cirrhosis (ICD 347); nephritis, nephritic syndrome and nephrosis (ICD 350); suicide (ICD E54); hypertensive disease (ICD 26); bronchitis, emphysema and asthma (ICD 323); septicaemia (ICD 038); tuberculosis (ICD 02); ulcers of the stomach and duodenum (ICD 341); certain conditions originating in the perinatal period (ICD 45); congenital anomalies (ICD 44); anaemias (ICD 200); homicide (ICD E55); meningitis (ICD 220); and protein-calorie malnutrition (ICD 192).

Demographic information was provided by the Ministry of Interior [15]. The smallest administrative units coded for examination of the various diseases cases or health care events were precincts and townships. Age-adjusted standard prevalence rates, a direct adjustment using the world population in 2000 as the standard population [16], was then calculated, the results showed the leading causes of death for males and females in each township.

### Global Moran's I statistic

The global spatial autocorrelation statistical method was used to measure the correlation among neighboring observations, to find the patterns and the levels of spatial clustering among neighboring districts [17]. The Moran's I statistic, which is similar to the Pearson correlation coefficient [18], is calculated by(1)

where *N *is the number of districts; *w*_*ij *_is the element in the spatial weight matrix corresponding to the observation pair *i*, *j*; and *x*_*i *_and *x*_*j *_are observations for areas *i *and *j *with mean *u *and(2)

Since the weights are row-standardized Σ*w*_*ij *_= 1, the first step in the spatial autocorrelation analysis is to construct a spatial weight matrix that contains information about the neighborhood structure for each location. Adjacency is defined as immediately neighboring administrative districts, inclusive of the district itself. Non-neighboring administrative districts are given a weight of zero.

### Determining spatial weights/connectivity matrices

Spatial contiguity for polygons is the property of sharing a common boundary or vertex. Contiguity analysis is an important method for assessing unusual features in the connectivity distribution [13,19]. The Queen's measure of contiguity can be utilized to make up for spatial contiguity by incorporating both the Rook and Bishop relationships into a single measure [19].

**Figure 2:**
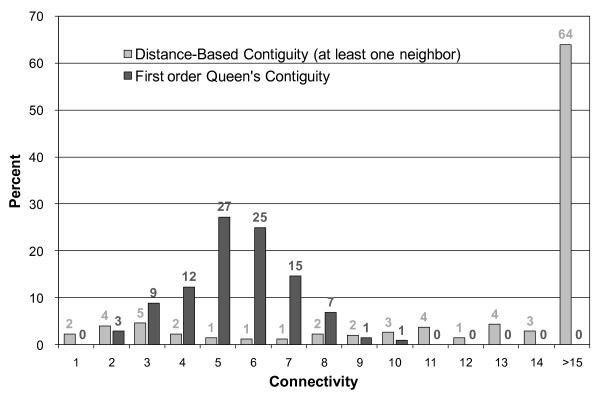
**Results of the analysis of the connectivity distributions of neighboring administrative district boundaries in Taiwan**.

The administrative districts considered in this study are highly irregular in both shape and size. We compare the first order queen polygon continuity method and a distance-based method, to choose the most appropriate method for quantifying the spatial weights matrix for analysis of the connectivity distributions between neighbors. Figure 2 shows the results of both the distance-based and the first order Queen's contiguity analysis for the administrative district boundaries. When the distance-based method is used there is a larger percentage of contiguity connection between neighbors (greater than 15); whereas the maximum value for the first order Queen's contiguity is 10. The differences between the distance-based contiguity and the first order Queen's contiguity methods are obvious. The connectivity distribution results obtained with the latter highlights the marked parities in connectivity. Based on the results of the connectivity distribution, we construct a first order queen polygon contiguity weight file for districts which share common boundaries and vertices. The spatial weights/connectivity matrices are utilized in the following local G*(d) calculations.

### Local *G*_i_*(d) statistic

The local *G*_i _*(d) statistic (local G-statistic) is used to test the statistical significance of local clusters (as related to the 20 leading causes of death), and to determine the spatial extent of these clusters [12,14]. The local G-statistic is useful for identifying individual members of local clusters by determining the spatial dependence and relative magnitude between an observation and neighboring observations [20]. The local G-statistic can be written as follows [12,21,22]:(3)

where *x *is a measure of the prevalence rate of each leading cause of death event within a given polygon (i.e., each administrative district); *w*_*ij *_is a spatial weight that defines neighboring administrative districts *j *to *i*; *W*_*i *_is the sum of the weights *w*_*ij*_, .

Developing the spatial weights *w*_*ij *_is the first step to calculating *G*_i_*(d). The spatial weight matrix includes *w*_*ij *_= 1. In this study, adjacency is defined using a first order queen polygon continuity weight file which has been constructed based on the districts that share common boundaries and vertices.

Non-neighboring administrative districts are given a weight of zero. The neighbors of an administrative district are defined as those with which the administrative district shares a boundary. A simple 0/1 matrix is formed, where 1 indicates that the municipalities having a common border or vertex; 0 otherwise [21,23].

The local G-statistic includes the value in the calculation at *i*. Assuming that *G*_i _*(d) is approximately normally distributed [12], the output of *G*_i _*(d) can be calculated as a standard normal variant with an associated probability from the z-score distribution [24]. Clusters with a 95 percent significance level from a two-tailed normal distribution indicate significant clustering spatially, but only positively significant clusters (the z-score value greater than +1.96) are mapped.

### Logistic regression analysis

Similarities between spatial distribution patterns for males and females are displayed. In addition to mapping, logistic regression is also performed. The binary response indicates whether there is significant autocorrelation between administrative districts or areas. There is higher correlation if the absolute value of the z-score of the local *G*-statistics is larger than 1.96; lower correlation otherwise. Gender is considered as an explanatory variable in the logistic regression model. Thus the model is expressed as(4)

where *β*_0 _and *β*_1 _are the logistic regression coefficients of the model. Pr(Higher correlation) and Pr(Lower correlation) denote the "Higher" and "Lower" correlation probabilities, respectively. Computation is performed with the R-language (R 2.8.1).

## Results

The results of the calculation of the global autocorrelation statistics for the top 20 leading cause of death events in the year 2006 in Taiwan are summarized in Table 1. The results of the global Moran's tests for all cases related to the leading causes of death (for both males and females) are statistically significant (z-score greater than 1.96) and indicate spatial heterogeneity.

**Table 1:** Global autocorrelation analysis of data for 20 leading health problems in Taiwan, 2006.

	Male		Female	
		
Leading cause-of-death events (ICD code)	Moran'I	Z(I)	Moran'I	Z(I)
Malignant neoplasms (ICD 08-14)	0.39	12.52	0.37	11.94
Cerebrovascular disease (ICD 29)	0.20	6.44	0.38	11.92
Heart disease (ICD 250, 251, 27, and 28*)	0.28	8.95	0.52	16.56
Diabetes mellitus (ICD 181)	0.25	8.17	0.38	12.13
Accidents and adverse effects (ICD E47-E53)	0.56	18.03	0.51	16.25
Pneumonia (ICD 321)	0.52	16.38	0.54	17.09
Chronic liver disease and cirrhosis (ICD 347)	0.27	8.91	0.50	16.02
Nephritis, nephritic syndrome and nephrosis (ICD 350)	0.39	12.50	0.23	7.27
Suicide (ICD E54)	0.36	11.38	0.20	6.39
Hypertensive disease (ICD 26)	0.55	17.22	0.62	19.52
Bronchitis, emphysema and asthma (ICD 323)	0.59	18.93	0.46	14.89
Septicaemia (ICD 038)	0.47	14.75	0.55	17.33
Tuberculosis (ICD 02)	0.42	13.16	0.56	18.10
Ulcer of stomach and duodenum (ICD 341)	0.30	9.39	0.56	17.67
Certain conditions originating in the perinatal period (ICD 45)	0.29	9.39	0.54	16.90
Congenital anomalies (ICD 44)	0.39	14.20	0.38	11.95
Anaemias (ICD 200)	0.21	6.58	0.48	15.27
Homicide (ICD E55)	0.14	6.27	0.35	11.67
Meningitis (ICD 220)	0.52	16.41	0.22	7.21
Other protein-calorie malnutrition (ICD 192)	0.32	10.16	0.14	6.21

Z(I) a value greater than 1.96 is statistically significant.

28* indicates the complete list including ICD codes 420-429.

**Figure 3:**
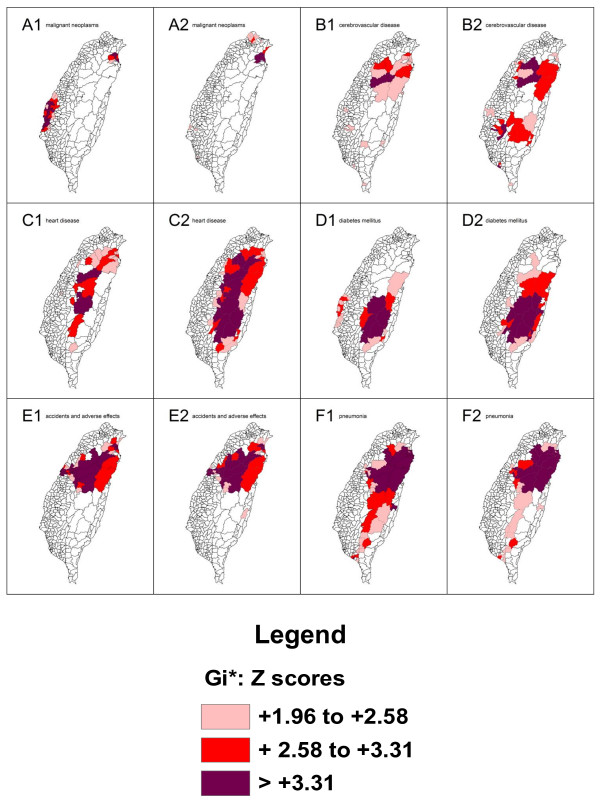
**Spatial clusters (hotspots) of the 20 leading causes of death from top 1 to 6 in Taiwan in 2006**. Maps showing the spatial clusters of the 20 leading causes of death from top 1 to 6 in Taiwan in 2006: malignant neoplasms are designated by A; cerebrovascular disease, B; heart disease, C; diabetes mellitus, D; accidents and adverse effects, E; pneumonia, F. Gender is indicated by a number, where male is 1 and female is 2.

**Figure 4:**
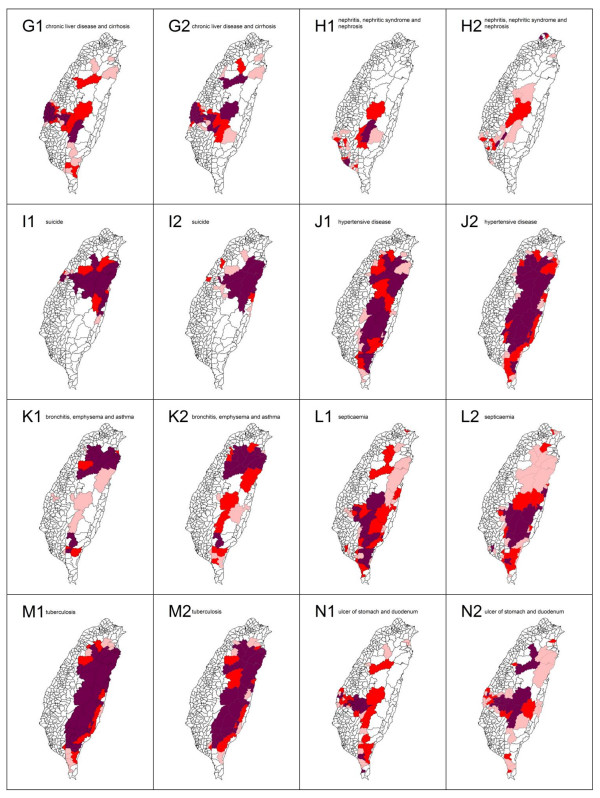
**Spatial clusters (hotspots) of the 20 leading causes of death from top 7 to 14 in Taiwan in 2006**. Maps showing the spatial clusters of the 20 leading causes of death from top 7 to 14 in Taiwan in 2006: chronic liver disease and cirrhosis are designated by G; nephritis, nephritic syndrome and nephrosis, H; suicide, I; hypertensive disease, J; bronchitis, emphysema and asthma, K; septicaemia, L; tuberculosis, M; ulcer of stomach and duodenum, N. Gender is indicated by a number, where male is 1 and female is 2.

**Figure 5:**
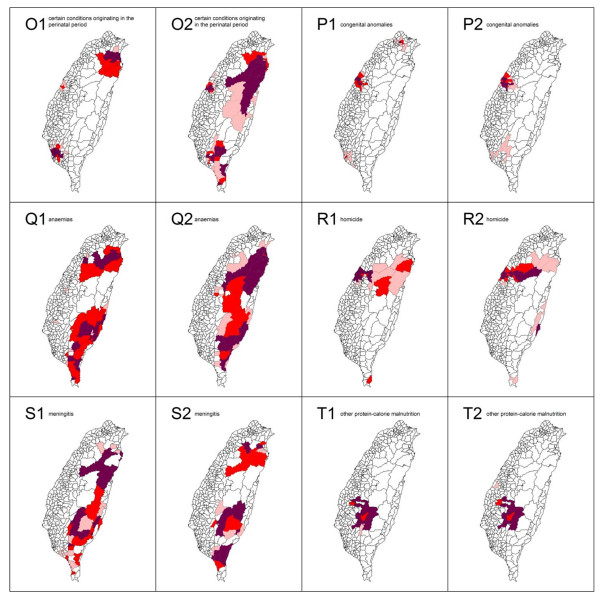
**Spatial clusters (hotspots) of the 20 leading causes of death from top 15 to 20 in Taiwan in 2006**. Maps showing the spatial clusters of the 20 leading causes of death from top 15 to 20 in Taiwan in 2006: certain conditions originating in the perinatal period are designated by O; congenital anomalies, P; anaemias, Q; homicide, R; meningitis, S; and other protein-calorie malnutrition, T. Gender is indicated by a number, where male is 1 and female is 2.

The spatial clusters (hotspots) as obtained from the local Gi*(d) statistic for the top 20 leading health care problems for both males and females in Taiwan in 2006 are shown in Figures 3, 4 and 5.

**Figure 6:**
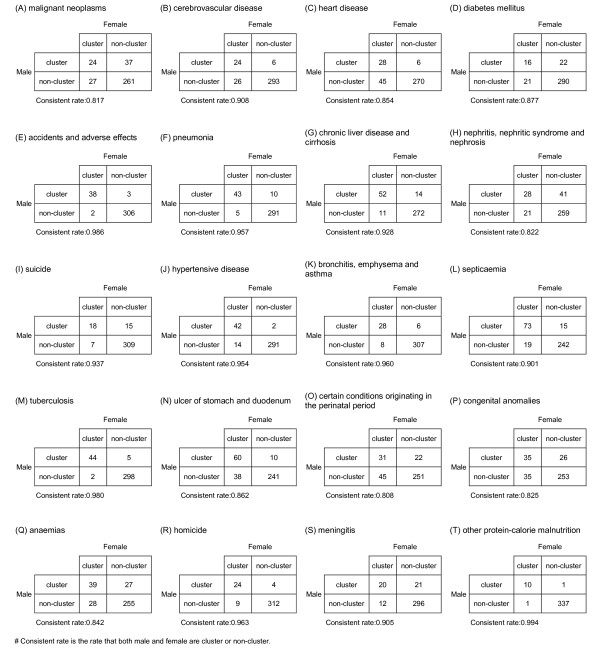
**Map of cross tabulations with consistency rates for the top 20 leading health care problems in Taiwan, 2006**.

The z-score outcomes as calculated by the Gi*(d) statistic are categorized as clusters or non-clusters, at the 5% significance level. This is followed by cross tabulation with the top 20 leading health problems. All results are summarized in Figure 6.

Furthermore, we find that there is no statistically significant dissimilarity (p-value > 0.05) between the spatial distribution patterns for males and females for fifteen out of twenty spatial clusters. We do find dissimilarities for cerebrovascular disease, heart disease, nephritis, nephritic syndrome and nephrosis, ulcers of stomach and duodenum, and certain conditions originating in the perinatal period. All results are shown in Table 2.

**Table 2:** Logistic regression model comparisons of the top 20 leading health problems in Taiwan by gender, 2006.

Leading cause-of-death event (ICD code)	p-value	description
Malignant neoplasms (ICD 08-14)	0.30	Similarity
Cerebrovascular disease (ICD 29)	0.02*	Dissimilarity
Heart disease (ICD 250, 251, 27, and 28*)	0.00***	Dissimilarity
Diabetes mellitus (ICD 181)	0.90	Similarity
Accidents and adverse effects (ICD E47-E53)	0.91	Similarity
Pneumonia (ICD 321)	0.59	Similarity
Chronic liver disease and cirrhosis (ICD 347)	0.77	Similarity
Nephritis, nephritic syndrome and nephrosis (ICD 350)	0.04*	Dissimilarity
Suicide (ICD E54)	0.27	Similarity
Hypertensive disease (ICD 26)	0.20	Similarity
Bronchitis, emphysema and asthma (ICD 323)	0.80	Similarity
Septicaemia (ICD 038)	0.73	Similarity
Tuberculosis (ICD 02)	0.74	Similarity
Ulcer of stomach and duodenum (ICD 341)	0.01*	Dissimilarity
Certain conditions originating in the perinatal period (ICD 45)	0.03*	Dissimilarity
Congenital anomalies (ICD 44)	0.38	Similarity
Anaemias (ICD 200)	0.92	Similarity
Homicide (ICD E55)	0.50	Similarity
Meningitis (ICD 220)	0.27	Similarity
Other protein-calorie malnutrition (ICD 192)	1.00	Similarity

*** Significance < 0.001; * significance < 0.05.

28* indicates the complete list included ICD codes 420-429.

## Discussion

Nearby locations are likely to possess similar attributes. In other words, everything is related to everything else, and nearby things are more closely related to nearby things than to distant things [25]. In epidemiology, a cluster becomes apparent when a number of health events occur which are situated close together in space and/or time. The evaluation of spatial distributions as a measure of disease risk may provide etiological insights [26]. Spatial autocorrelation is defined as the relation between the values of a single variable. This relation is attributable to the geographic arrangement of areal units on a map and can be used to identify the degree of spatial clustering [27,28]. In this study, the local G-statistic is used to measure the degree of spatial clustering and map the geographic patterns of the areal units. Spatial clustering of the leading cause of death (also called a hot spot) is defined as when we obtain z-score values larger than 1.96. In epidemiology, hot spots are considered interesting because of their correlation to etiology. For this reason, we indicate the hot spots of 20 leading cause of death, as obtained from our analysis, and identify their spatial locations. Information about spatial location is useful for detecting risk factors from a spatial viewpoint. A more detailed survey of these identified hot spots may reveal important clues as to risk factors for these diseases.

To appropriately use public health data aggregated according to irregular administrative districts it is important to decide on the local measures of spatial autocorrelation for the specification of local neighborhood (as defined by the spatial weights matrix). In general, the spatial autocorrelation may be the strongest between the nearest neighbors. As the neighborhoods increase in number, this autocorrelation weakens [29]. A formal guidance for choosing a proper spatial weight matrix has not yet been developed [30,31]. Therefore, the proper spatial weight matrix is chosen after a comparison of the connectivity distributions of neighbors obtained with the distance-based contiguity and the first order Queen's contiguity methods. However, an evaluation of the sensitivity of the results to the different spatial weight matrices still needs to be developed and assessed for further studies.

The modifiable areal unit problem (MAUP) is a phenomenon whereby different results are obtained from analysis of the same data, grouped into different sets of areal units. The MAUP can be subdivided into two separate effects that usually occur simultaneously during the analysis of aggregated data. The scale effect causes variation in statistical results given different levels of aggregation. In other words, association between variables depends on the size of the areal units for which data are reported. Generally, correlation increases as the size of the areal unit increases. The zone effect describes variation in correlation statistics caused by the regrouping of data into different configurations but with the same scale. These effects occur because spatial processes generating the observed data may exist at scales and for particular areal units that may be reflected more or less accurately by the boundaries in use [32]. Studies of the MAUP based on empirical data provide only limited insight of the inability to control relationships between multiple spatial variables. Data simulation is necessary to control over various properties of individual level data. Simulation studies, such as those by Swift et al. (2008), have demonstrated that the spatial support of variables can affect the magnitude of the ecological bias caused by spatial data aggregation [33]. Manley et al. (2006) concluded that MAUP is not really a problem but in constrast, a resource. Data at different scales can help us identify processes operating at different scales. It is clear that it is not possible to define an ideal single census geography that captures all the processes for all variables [32]. Furthermore, the internal composition of the areal units may not be homogeneous, particularly for disease distribution. Further to this, Matisziw et al. (2008) suggested that downscaling the spatial structure of polygonal units sould provide valuable information on the spatial distribution of disease [34].

This is the first study of the spatial distribution of the 20 leading health problems in Taiwan. There have been few previous ecological studies related to health care issues and their correlation to risk factors in Taiwan, although malignant neoplasms and tuberculosis have been documented and are discussed briefly below. We hope that this study of the spatial clustering of Taiwan's leading health issues can provide help for the study of spatial epidemiology.

Residents along the southwestern and northeastern coasts of Taiwan drank well water contaminated with a high concentration of arsenic before the establishment of the public water system [35]. Residents in these areas were found to have an increased risk of malignant neoplasms, including cancers of the liver, nasal cavity, lung, skin, bladder and kidney, for both males and females, as well as prostate cancer in males [36,37]. Although well water was no longer used for drinking or cooking after the mid-1970s, there was still significantly increased risks of urinary cancers [38,39] and lung cancer [39,40] in the arseniasis-endemic areas of southwestern and northeastern Taiwan. Our results, showed clusters for malignant neoplasms in these arseniasis-endemic areas, but also did reveal a new carcinogen clustering (for females) in the northen coastal region of Taiwan. This is worthy of more investigation in the future.

According to data from the Center for Disease Control in Taiwan, there is a four-fold higher incidence of tuberculosis in aboriginal portions of the population than in people of Han ethnicity (Hans) [41]. Environmental factors such as hygiene, income, and social behavior (e.g., alcoholism) have been blamed for the prevalence of tuberculosis in aboriginal populations. Genetic variations in NRAMP 1 may also affect susceptibility to and increase the risk of tuberculosis in Taiwanese aboriginals [42]. Here we calculate tuberculosis clusters for males and females by utilizing the local G-statistic. The results show clear spatial clustering in Taiwanese aboriginal townships. Thus, our observations support the results obtained in previous studies. In addition, the hypertensive disease cluster, also possibly closely correlated to mountainous and aboriginal townships, is also worthy of attention. The strength of the relationship between aboriginal populations and hypertensive disease clusters needs further study to clarify. A more detailed survey of hypertensive disease may reveal valuable findings in terms of the risk factors between populations (four main populations are distributed in Taiwan) and hypertensive disease.

The z-scores for the local G-statistic are calculated using the logistic regression model. The results for various leading health problems and gender are compared. The test results show statistically significant differences for five health care problems in Taiwan in the year 2006, but another fifteen cases which are not, on the other hand. In other words, the null hypothesis is accepted. The accepted null hypothesis results indicate that the common spatial factor(s) may interact with both sexes.

## Conclusions

Spatial autocorrelation calculation is useful for cluster mapping of regional health care problems. Cluster mapping helps to clarify issues such as the spatial aspects of both internal and external correlations of leading health care events. This helps planners to assess spatial risk factors, and to ascertain what would be the most advantageous types of health care policies for the planning and implementation of health care services. These issues can greatly affect the performance and effectiveness of health care services and also provide a clear outline for helping us to better understand the results in depth.

## Competing interests

The authors declare that they have no competing interests.

## Authors' contributions

PJT conceived of the study, and participated in its design and coordination. MLL and CMC participated in the technical support of the ArcGIS program. CHP performed the statistical analysis.

## Pre-publication history

The pre-publication history for this paper can be accessed here:

http://www.biomedcentral.com/1471-2458/9/464/prepub
